# Step Sizes and Rate Constants of Single-headed Cytoplasmic Dynein Measured with Optical Tweezers

**DOI:** 10.1038/s41598-018-34549-7

**Published:** 2018-11-05

**Authors:** Yoshimi Kinoshita, Taketoshi Kambara, Kaori Nishikawa, Motoshi Kaya, Hideo Higuchi

**Affiliations:** 1 Department of Physics, Graduate School of Science, The University of Tokyo, 7-3-1 Hongo Bunkyo-ku, Tokyo, 113-0033 Japan; 2 Present Address: Center for Biosystems Dynamics Research, RIKEN, 6-2-3 Furuedai, Suita, Osaka 565-0874 Japan

## Abstract

A power stroke of dynein is thought to be responsible for the stepping of dimeric dynein. However, the actual size of the displacement driven by a power stroke has not been directly measured. Here, the displacements of single-headed cytoplasmic dynein were measured by optical tweezers. The mean displacement of dynein interacting with microtubule was ~8 nm at 100 µM ATP, and decreased sigmoidally with a decrease in the ATP concentration. The ATP dependence of the mean displacement was explained by a model that some dynein molecules bind to microtubule in pre-stroke conformation and generate 8-nm displacement, while others bind in the post-stroke one and detach without producing a power stroke. Biochemical assays showed that the binding affinity of the post-stroke dynein to a microtubule was ~5 times higher than that of pre-stroke dynein, and the dissociation rate was ~4 times lower. Taking account of these rates, we conclude that the displacement driven by a power stroke is 8.3 nm. A working model of dimeric dynein driven by the 8-nm power stroke was proposed.

## Introduction

Cytoplasmic dynein is a microtubule-based motor protein that transports intracellular cargos and regulates mitosis in cells^1^. Dynein converts the energy liberated from ATP hydrolysis into mechanical motion. Dynein moves toward minus end of microtubule with the step sizes of ~8 nm and multiple of 8 nm measured by single molecular methods^2–6^. The step size was determined by the spacing of 8-nm between tubulin dimers. It is proposed that the directionality of dynein stepping is determined by the direction of power stroke of dynein linker. However, a size of the power stroke measured by the single molecular methods had not been reported in spite that the size is important to understand the stepping mechanism.

The size of the power stroke has been investigated by the structural studies^7–11^. In the absence of microtubules, the position of the microtubule binding domain (MTBD) relative to the tail domain of dynein was changed by ~15 nm from the ADP-Vi to the apo states^7,8^ (Table S1). Three-dimensional electron microscopy structures of axonemal dynein in axoneme were reconstructed, and the ring domain and MTBD of dynein were shifted by 5–12 nm relative to the tail domain along the microtubule from the prestroke to poststroke states^9–11^ (Table S1). Although an electron microscope is a powerful method for observing the shift, there are several problems with these studies. The moving distance could not be measured precisely, because large parts of the structures of the stalk and MTBD were invisible due to their large flexibility. Additionally, a nonphysiological analog (ADP-Vi) was used to make the pre-stroke state, and the static images could not exactly determine the sequential changes of the structure along the microtubule. It is therefore crucial to obtain the displacement of dynein interacting with the microtubule under physiological conditions.

To understand the mechanochemical reaction of human cytoplasmic dynein, we investigated the displacement of microtubules driven by the power stroke of a single-headed dynein by optical tweezers. The mean displacement was ~8 nm at high ATP concentration and decreased sigmoidally with the decrease in ATP concentration. The efficiency of fluorescence resonance energy transfer (FRET) in a single-headed dynein also depended on the ATP concentration. The ATP dependence of the mean displacement was explained by a mechanochemical model. The model was verified quantitatively by the calculation using kinetic rates that were measured by the dwell time and biochemical assay. From the results of 8-nm displacement and the chemical rates, we proposed a walking model of dimeric dynein.

## Results

### Constructs of single-headed dynein molecules and FRET efficiency

**Figure 1:**
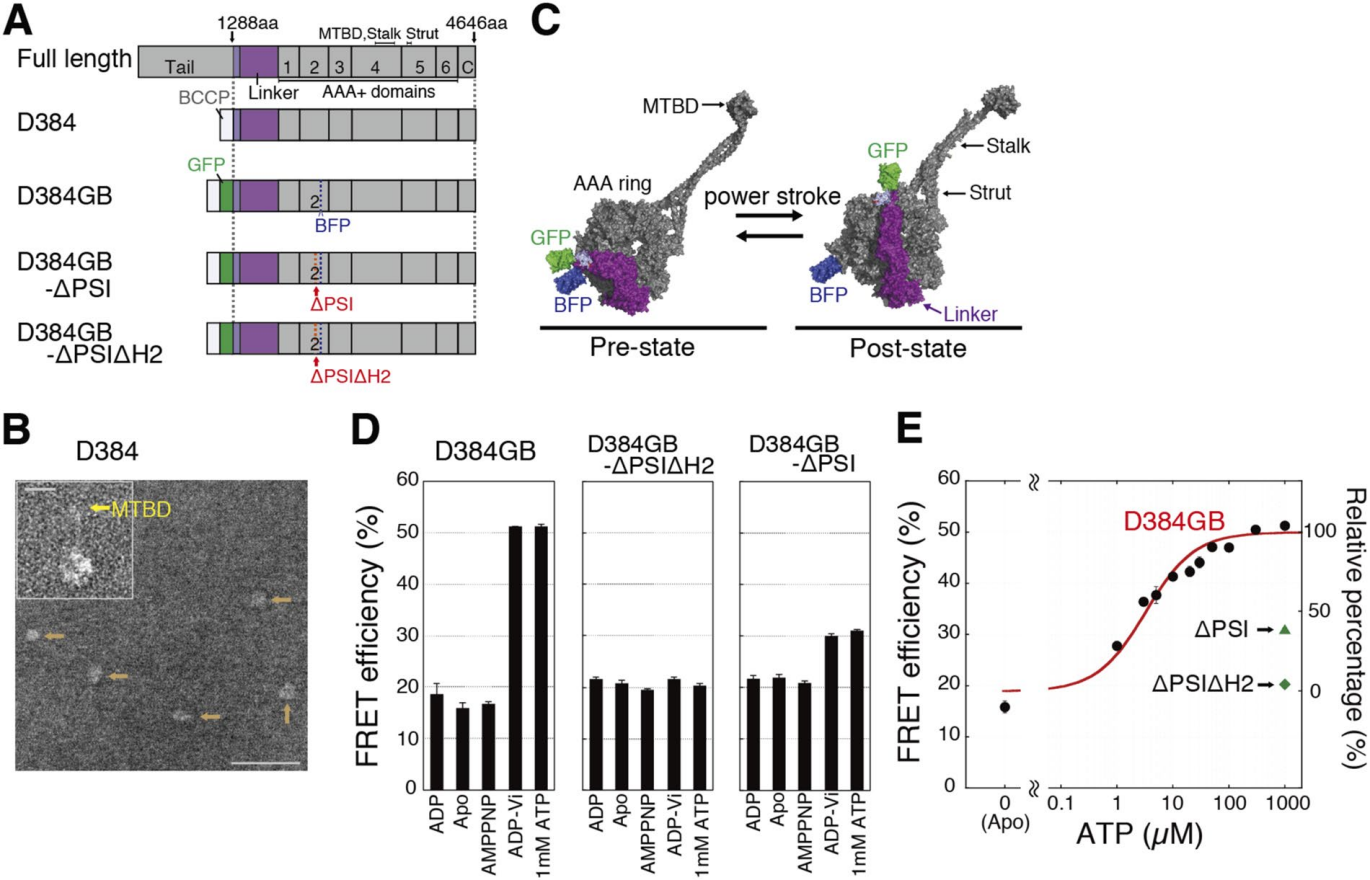
FRET measurements of dynein. (**A**) Schematics of the dynein constructs. (**B**) Electron microscope image of dynein molecule D384, marked with orange arrows. The MTBD in the magnified image of dynein is marked with a yellow arrow in the inset. Scale bars, 60 nm and 10 nm (inset). (**C**) Structures of the dynein motor domain (D384GB, PDB 4RH7 and 3VKH) before and after the power stroke. BFP and GFP are bound to the ring and linker, respectively. (**D**) FRET efficiencies of D384GB, D384GB-ΔPSI and D384GB-ΔPSIΔH2 in various chemical states. (**E**) ATP dependence of the FRET efficiencies of D384GB. The efficiencies of D384GB were fit to a sigmoidal curve (red line, curve equation 5, described in the Supplemental Information). At 3.3 μM ATP, the relative percentage of population taking the highest FRET efficiency was half. The R^2^ was 0.97. Error bars in (**D**) and (**E**) represent the standard error.

We constructed a single-headed dynein, D384, of the human cytoplasmic dynein motor domain for the optical tweezer experiments (Fig. 1A). This construct was confirmed to be monomeric by electron microscopy (Fig. 1B). To detect the power stroke of dynein, we constructed D384GB, in which BFP and GFP were fused to the AAA2 domain and the N-terminus of the linker in D384, respectively (Fig. 1A and C). To understand the contribution of the power stroke to the displacement of dynein, we made nonstroking mutants, D384GB-ΔPSIΔH2 and -ΔPSI (Fig. 1A, see Materials and Methods). PSI and H2 loops in the AAA2 domain interacted with the kinked linker (Fig. S1A). By the deletion of these loops, dynein took the poststroke configuration even in the presence of ADP-Vi^12,13^.

The FRET efficiencies between BFP and GFP in D384GB were measured to investigate the linker swing of dynein in the absence of microtubules. The efficiencies were low (~17%) in the poststroke states (apo, ADP and AMPPNP) and high (51%) in the prestroke states (ADP-Vi and 1 mM ATP) (Fig. 1D), confirming that the dynein linker would be straight in the poststroke state and kinked in the prestroke state^14^ (Fig. 1C). With an increase in ATP concentration, the efficiency increased sigmoidally from 17 to 51% (Fig. 1E).

**Figure 2:**
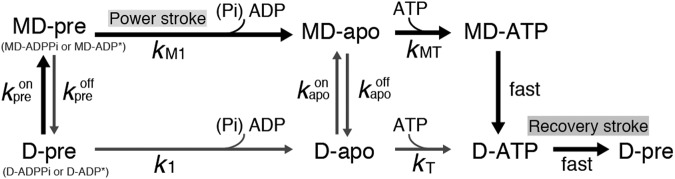
Biochemical reaction model of dynein with (upper row) and without (lower row) microtubules. The main route of the ATPase cycle in the presence of a microtubule at high ATP concentration is shown by bold arrows. MD-pre and D-pre, prepower stroke states (chemical state of pre states has not identified whether ADPPi or ADP* state); MD-apo and D-apo, no-nucleotide binding states; MD-ATP and D-ATP, ATP binding states. The superscripts of the reaction rates were partly defined by their association (on) or dissociation (off) from microtubules. The subscripts were defined as the chemical or structural states or binding of microtubules. For more details, see the text.

To explain the ATP dependence of the FRET efficiency, we simplified the chemical reaction model of dynein without microtubules (lower row in Fig. 2). The reaction states in the model were the ATPase states in the AAA1 catalytic site, because the linker swing and activity of dynein were predominantly coupled to ATP binding and hydrolysis at the AAA1 site^14,15^. The rate (*k*_1_) from the prestroke to the apo state includes the power stroke and product release and *k*_T_ is the ATP binding rate (Fig. 2). In this model, faster reaction rates and reverse rates of the dynein ATPase cycle were neglected under our experimental conditions at <100 μM ATP and in the absence of ADP and Pi^16–20^. The efficiency was then fitted with the modified Michaelis-Menten equation at *k*_1_/*k*_T_ (=*K*_m_^E^) of 3.3 ± 0.3 μM (mean ± s.e.m.) (Fig. 1E and equation 5 in the Supplemental Information). These results suggest that the population of dynein in the prestroke and poststroke states, respectively, increases and decreases with the increase of the ATP concentration in the absence of microtubules.

### Displacement of single-headed dynein

**Figure 3:**
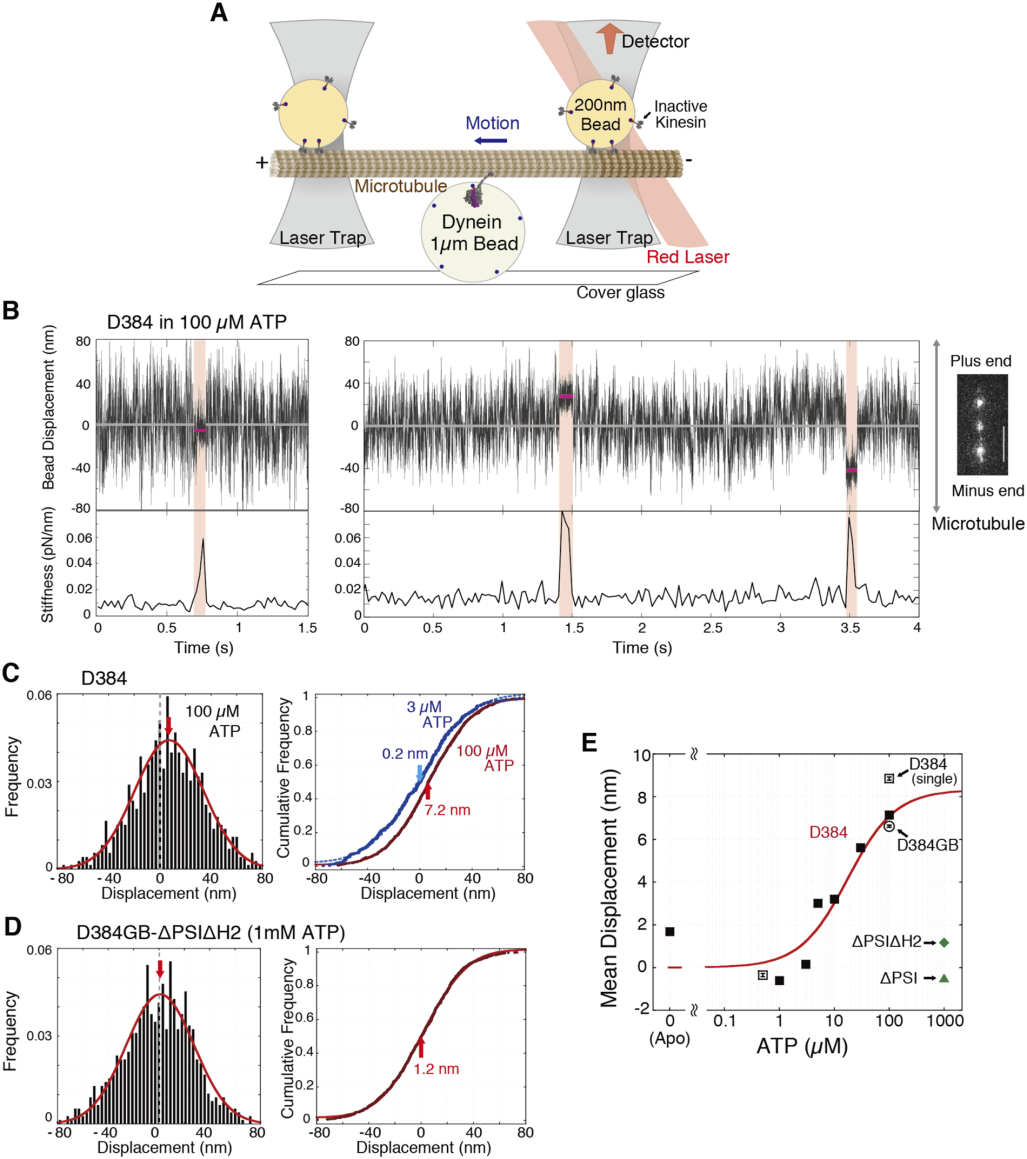
Measurements of displacement of single-headed dynein. (**A**) Schematic diagram of the dumbbell assay. (**B**) Time traces of position along the microtubule of the trapped bead. The red zones and lines indicate the period of dynein binding to the microtubule at higher stiffness (or lower variance) and the displacement during the period. Right panel, fluorescence microscope image of polarity-marked microtubule and beads. Scale bar, 5 µm. (**C**,**D**) Distributions of displacements of D384 at 3 µM ATP (blue in C, 494 events, 5 dynein-beads) or 100 µM ATP (red in C, 727 events, 7 dynein-beads) and those of D384GB-ΔPSIΔH2 at 1 mM ATP (773 events, 10 dynein-beads) were measured by the dumbbell assay. Left panels show the Gaussian distributions of the displacements. Right panels show the mean displacements determined by fitting experimental displacements to the integrated Gaussian function, shown by blue and red lines. Arrows show the mean displacements. (**E**) Mean displacements measured by the single-trap of D384 (open squares), dumbbell assays of D384 (closed squares), D384GB (open circle), and the mutants (closed diamond and triangle). Most of the standard error bars are within the symbols. The mean displacements of D384 were fit to the equation (red line, curve equation 6, described in the Supplemental Information). The R^2^ was 0.977.

The displacement of dynein interacting with microtubules was detected by dumbbell optical tweezers under no load (Fig. 3A). The biotin in the N-terminus of the dynein construct was bound to an avidin-coated larger bead that was fixed on the glass surface. Both ends of polarity-marked microtubules were bound to smaller beads via inactive kinesin molecules. The microtubules were then brought into contact with the dynein-coated beads. Dynein bound randomly to the thermally moving microtubule-beads and then generated the displacement, as observed in the single-skeletal myosin-S1 measurement^21^. Therefore, the measured displacement was the sum of the displacement driven by dynein and the thermal displacement of the microtubule^21^.

We determined the period of dynein-microtubule binding in which the Brownian noise of the trapped beads was decreased (red zones in Fig. 3B). Dynein took single steps immediately after binding to microtubules and dissociated from the microtubules without exhibiting further movement. We analyzed the displacement of the microtubule in the binding period and the time of dynein binding to the microtubule. The histograms of the displacements, therefore, fit well to single Gaussian distributions, with the shifts corresponding to the mean displacement (left panel in Fig. 3C). The displacements were broadly distributed with a large standard deviation, because the Brownian motion of the microtubule-beads was large at low trap stiffness^21^. To accurately determine the mean displacements, we analyzed them by fitting the cumulative distributions of the displacements to the curves calculated by integrating the Gaussian function (right panel in Fig. 3C, equation 2 in the Supplemental Information). At an ATP concentration of 100 μM, the microtubule was driven by dynein D384 with a mean displacement of 7.2 nm toward the plus end (in other words, dynein moved by 7.2 nm toward the minus end of the microtubule) (Fig. 3B and C).

### Displacement of nonstroking mutant dynein

To understand the contribution of the powerstroke to the step, we measured the step sizes and FRET efficiencies of the nonstroking mutants D384GB-ΔPSIΔH2 and -ΔPSI. The FRET efficiencies of the ΔPSIΔH2 and ΔPSI mutants in the presence of 1 mM ATP and ADP-Vi were as low as those in the other nucleotide condition (Fig. 1D and E), indicating that the linkers of the mutants maintained the straight form. Both mutants bound to microtubules, but did not show microtubule gliding activity at all (data not shown). The mutants bound strongly to microtubules, and the mean displacements of the mutants were close to zero, 1.2 ± 0.1 (D384GB-ΔPSIΔH2) and −0.5 ± 0.1 nm (D384GB-ΔPSI), at 1 mM ATP (Figs 3D and E). In contrast to the mutants, the mean displacement at 100 μM ATP of D384GB without the deletions of loops was 6.6 ± 0.1 nm, which was close to the mean displacement of D384 at the same ATP concentration (Fig. 3E and S2A). These results indicate that mutant dynein molecules neither generate a displacement of microtubules nor swing the linker. Thus, the steps should be driven by the power stroke of D384 (or D384GB).

### ATP-dependent displacement of dynein

From the results of the FRET experiments, dynein in the prestroke and poststroke states was predominant at high and low ATP concentrations, respectively; therefore, the observed mean displacement of D384 should depend on the ATP concentration. The mean displacements at the low concentration of ATP (0–3 μM) were close to 0 nm (Fig. 3E). This indicates that dynein in the apo (or poststroke) state is predominant at low ATP concentrations in the FRET measurements, so that dynein in the apo state binds to microtubules and then dissociates from the microtubules neither a conformational change of the linker nor a stepping motion. The observed mean displacement of single-headed dynein increased sigmoidally to 7.2 nm as the ATP concentration increased (Fig. 3E), suggesting that the population of dynein that binds to microtubules in the prestroke state and generates the power stroke increased with the ATP concentration.

In the dumbbell method, dynein interacted in random orientations with microtubules because dynein bound randomly to the beads fixed on the glass surface. The angle between dynein and the microtubule might affect the displacement, as it was reported that the mean displacement of myosin depends on the angle between myosin and the actin filament^22^. To examine whether the displacement depends on the orientation of dynein to the microtubule axis, we measured the displacement of D384 bound on single trapped beads, thereby allowing dynein to interact with microtubules in the appropriate orientation due to the free rotation of the beads (Fig. S2B). The mean displacements were −0.4 ± 0.1 and 8.9 ± 0.1 nm at ATP concentrations of 0.5 and 100 μM, respectively (Figs 3E and S2B), which were close to those obtained by the dumbbell method. These results suggest that dynein mainly binds to microtubules in the appropriate orientations in the dumbbell method and that the linkage between dynein and the beads is so flexible that the linker swing of dynein is not amplified by the motion of the trapped bead rotated around the linkage.

### Calculation of ATP-dependent displacement

Here, we constructed the chemical reaction model of dynein in the presence of microtubules (Fig. 2). In the model, dynein binds to microtubules in a prepower stroke state, takes a power stroke, releases products (Pi and ADP), and then transits to the MD-apo state. It is presumed that the chemical state just before the power stroke are ADPPi or ADP* states, but there is no direct evidence whether the power stroke of cytoplasmic dynein is generated before or after phosphate release at the binding to microtubule^17,18,20^. Therefore, in this model, we defined the prestroke state bound to microtubule as the MD-pre that is structural state but not chemical state. The rate constant (*k*_M1_) from MD-pre to MD-apo includes the rates of the power stroke and the release of Pi and ADP. Dynein at MD-apo consecutively binds ATP (MD-ATP) or dissociates from microtubules at MD-apo before ATP binding. At lower ATP concentrations, most dynein predominantly binds to microtubules in the D-apo state and then dissociates from microtubules before or after binding ATP. The dissociation rate from MD-apo to D-apo was defined as *k*_apo_^off^, and the second-order rate constant of ATP binding from MD-apo to MD-ATP was defined as *k*_MT_. We assumed that the rate constant from MD-ATP to D-ATP is neglected, because it was much higher than that from MD-apo to MD-ATP under our experimental condition at < 100 μM ATP^23^.

The population of dynein with the power stroke decreases with the decrease in the ATP concentration, as mentioned above. Therefore, the distribution of experimental displacements should involve two Gaussian distributions with the stroke distance (~8 nm, obtained later) and with no displacement (0 nm). However, the histograms of the displacement fit well to a single Gaussian distribution, because the stroke distance (~8 nm) was much smaller than the standard deviation (>20 nm) of the distributions derived from Brownian motion (Figs 3C,D and S2A). Actually, the sum of the two Gaussian distributions simulated at a standard deviation of 20 nm and was not significantly different from a single Gaussian distribution (Fig. S3A and B), indicating that the distributions of the experimental data are consistent with the sum of a double Gaussian distribution.

We obtained the relationship between the calculated mean displacement and the ratio (*R*_M1_) of the number of power-stroked dynein molecules to total dynein molecules. The mean displacement was approximately directly proportional to the ratio (*R*_M1_) (Fig. S3C); therefore, the mean displacement was calculated from the power stroke size multiplied by the ratio (see details in the Supplemental Information). The mean displacement fit well to the modified Michaelis-Menten equation (Fig. 3E, equation 6 in the Supplemental Information). The power stroke size at saturated concentrations of ATP was calculated to be 8.3 ± 0.3 nm, and the ATP concentration at the halfway point of the size was 17.6 ± 0.5 μM (*K*_m_^d^ = *K*_m_^E^*k*_apo_^on^/*k*_pre_^on^) (Fig. 3E). The binding rate (*k*_apo_^on^) of dynein in the D-apo state was 5.3 ± 0.5 times of that (*k*_pre_^on^) in the D-pre state (Fig. 2). These data suggest that a stroke size of 8.3 nm was generated by dynein binding to microtubules in the D-pre state and that the population of dynein taking the 8.3 nm step in the power stroke increased with the increase of the ATP concentration.

### Rate constants of the dynein ATPase reaction

**Figure 4:**
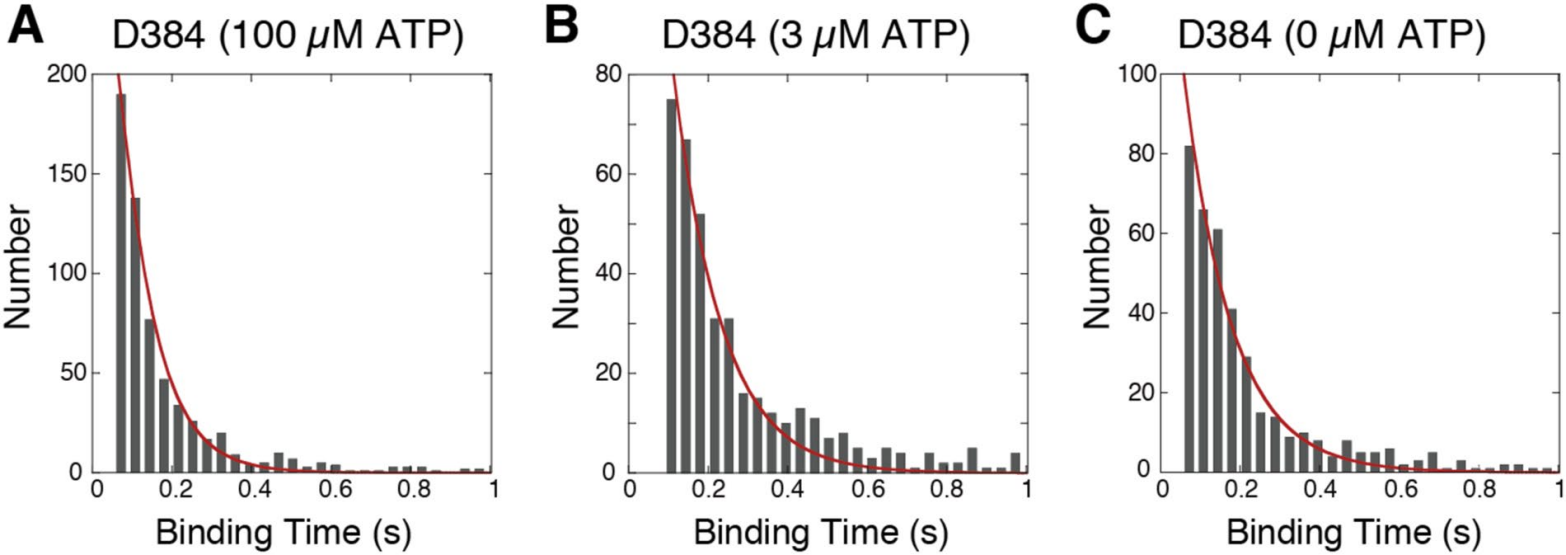
Distributions of microtubule binding time of D384 at 100 μM (A, 727 events, 7 dynein-beads), 3 μM (B, 494 events, 5 dynein-beads) and 0 μM ATP (C, 436 events, 6 dynein-beads). Distributions were globally fit to the equation (red lines, curve equation 10, described in the Supplemental Information). The R^2^ of the fittings were 0.97, 0.96 and 0.97 at ATP concentration of 100, 3, and 0 μM, respectively.

To understand the kinetic rates in the presence of microtubules, we analyzed the time of dynein binding to microtubules. The binding time at 100 μM ATP was slightly longer than that at 3 μΜ ATP (Figs 4A,B and S2C). In the absence of ATP, the distribution of the binding time was similar to that at 3 μΜ ATP (Fig. 4B and C). This result implies that most of the dynein molecules that bind to microtubules in the D-apo state take the dissociation reaction from the MD-apo to D-apo state at low ATP concentrations.

We calculated the rate constants (*k*_M1_, *k*_MT_ and *k*_apo_^off^), shown in Fig. 2, from the distribution of the binding time, which was dependent on the ATP concentration. There are two routes that dynein bound to a microtubule can dissociate from it in the chemical model (Fig. 2). In one route, dynein in the D-pre state binds to microtubules (MD-pre), transits to the MD-apo state and then consecutively binds ATP (MD-ATP) or dissociates from microtubules at MD-apo before ATP binding (the binding time was explained with the equation 7 in the Supplemental Information). In the other reaction route, dynein in the D-apo state binds to microtubules (MD-apo) and then binds to ATP (MD-ATP) or dissociates from microtubules before ATP binding (D-apo) (the binding time was explained with the equation 9 in the Supplemental Information). The total binding distribution including the two routes (equation 10 in the Supplemental Information) is given by summing equations 7 and 9, weighted by the number of dynein molecules that take each route.

All of the histograms were fit globally by equation 10 (solid lines in Figs 4 and S2C). The best parameter set (*k*_M1_, *k*_MT_ and *k*_apo_^off^) was 12 ± 1 s^−1^, 0.29 ± 0.10 μM^−1^ s^−1^ and 8.3 ± 0.4 s^−1^, respectively, with a mean square (R^2^ = 0.95) of regression errors. The mean binding time calculated from equation 10 was not strongly dependent on the ATP concentration (Fig. S3D and equation 11 in Supplemental Information).

### ATPase activity and affinity of dynein-microtubule complex

To evaluate the obtained rate constants and estimate the other constants shown in Fig. 2 and Table 1, the ATPase rate and dissociation constant of single-headed dynein were measured by a biochemical assay. The ATP turnover rates were measured by the concentration of released inorganic phosphate. The maximum turnover rates and Michaelis-Menten constants in the absence of microtubules were 0.40 ± 0.01 s^−1^ (*k*_basal_) and 31 ± 4 μM (*K*_m_^ATP^) for D384 (Fig. S4), and 0.17 ± 0.004 s^−1^ and 27 ± 3 μM for D384GB (data not shown), indicating that the Michaelis-Menten constants of D384 and D384GB are approximately equal. The turnover rate and Michaelis-Menten constant of D384 in the presence of microtubules were 9.3 ± 2.5 s^−1^ (*k*_cat_) and 50 ± 21 μM (*K*_m_^MT^), respectively (Fig. S4). The ATPase activity of D384 increased by approximately 23-fold in the presence of microtubules. The high *K*_m_^MT^ value showed the low affinity of single-headed dynein to microtubules.

**Table 1:** List of kinetic constants of single-headed dynein.

Kinetic constants	Values
*k*_1_/*k*_T_ (=*K*_m_^E^)	3.3 ± 0.3 μM
*k*_apo_^on^/*k*_pre_^on^(=*K*_m_^d^/*K*_m_^E^)	5.3 ± 0.5
*k*_M1_	12 ± 1 s^−1^
*k*_apo_^off^	8.3 ± 0.4 s^−1^
*k*_MT_	0.29 ± 0.10 μM^−1^s^−1^
*K*_*d*_^*apo*^ (=*k*_apo_^off^/*k*_apo_^on^)	1.5 ± 0.1 μM
*K*_*d*_^*Vi*^ (~ *k*_pre_^off^/*k*_pre_^on^)	34 ± 3 μM
*k*_basal_	0.40 ± 0.01 s^−1^
*k*_cat_	9.3 ± 2.5 s^−1^
*K*_m_^ATP #^	31 ± 4 μM
*K*_m_^MT^	50 ± 21 μM
*k*_apo_^on^*	5.5 ± 0.5 μM^−1^s^−1^
*k*_pre_^on^ *	1.0 ± 0.1 μM^−1^s^−1^
*k*_pre_^off^ *	34 ± 5 s^−1^

^#^In the absence of microtubule.

*The rate constancts *k*_apo_^on^, *k*_pre_^on^, *k*_pre_^off^ were calculated from *k*_apo_^off^ divided by *K*_*d*_^*apo*^, *k*_apo_^on^ divided by *k*_apo_^on^/*k*_pre_^on^ and *K*_*d*_^*Vi*^ divided by obtained *k*_pre_^on^, respectively.

The dissociation constants (*K*_d_^apo^ and *K*_d_^Vi^) of D384 from microtubules in the apo and ADP-Vi states were calculated from the concentration of dynein in the supernatant after centrifugation of the dynein-microtubule complex. The calculated *K*_d_^apo^ and *K*_d_^Vi^ were 1.5 ± 0.1 μM and 34 ± 3 μM, respectively (Fig. S5). Dynein bound strongly to microtubules in the apo-state and weakly in the ADP-Vi state.

### Force, dwell time, velocity and the duty ratio of single molecules of dimeric dynein

**Figure 5:**
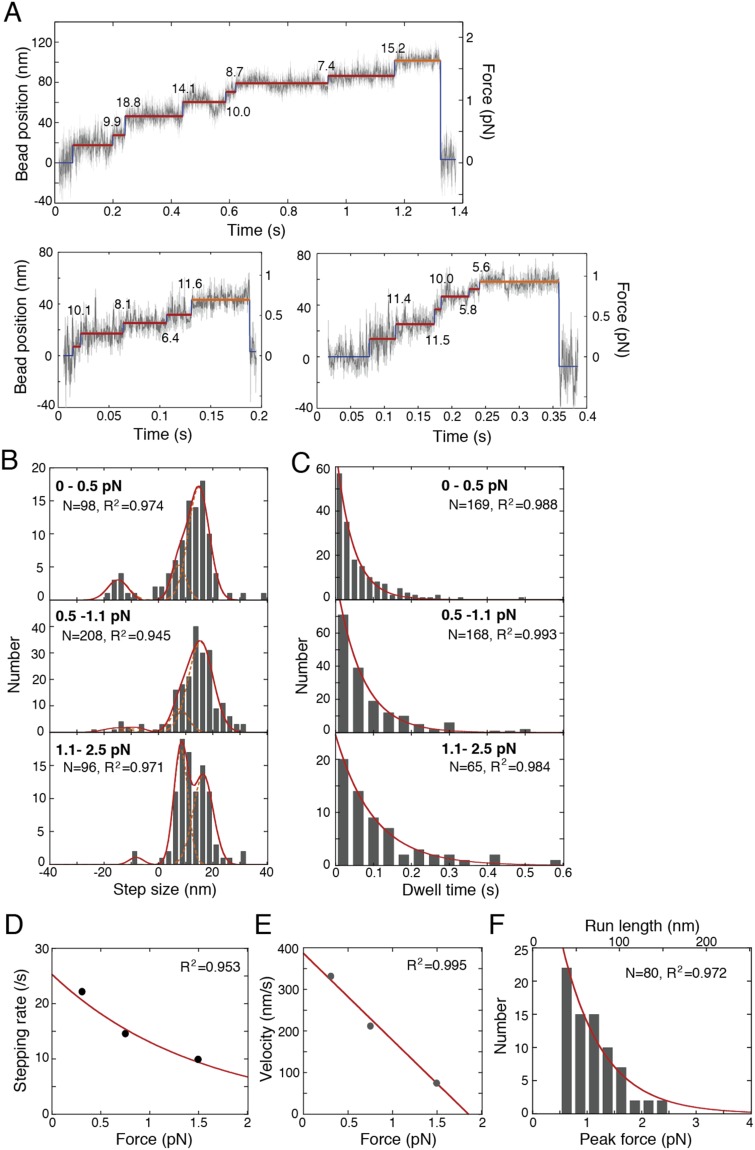
Movement and force generation of single molecules of dimeric dynein (GST-D384). (**A**) Typical displacement of the trap bead toward the minus end of the microtubule at 1 mM ATP. Red lines: dwell time analyzed by the step-finding algorithm in figure 5D. Orange lines: peak force immediately before dynein dissociates from the microtubule. Numbers in figures: the step size was analyzed in figure 5B. (**B**) The histograms of the step size were fit to multiple Gaussian distributions. Over a 1.1 pN force, the step size was calculated to be 8.23 ± 0.09 nm, 16.26 nm and −8.23 nm. (**C**) The histograms of the dwell time were fit to a single-exponential curve with a constant of 45 ± 1 ms (<0.5 pN), 69 ± 1 ms (0.5–1.1 pN) or 101 ± 1 ms (>1.1 pN). (**D**) The stepping rate depending on the force was fitted to a single-exponential curve. The maximum stepping rate under no load was 25.3 ± 0.2 s^−1^. (**E**) Relationship between force and velocity. The velocity was calculated as the stepping rate multiplied by the mean step size. The stall force and maximum velocity were calculated to be 1.85 ± 0.01 pN and 387 ± 5 nm/s, respectively, by fitting the data with a linear approximation. The error bars in D and E are almost within the symbols. (**F**) The histogram of the peak force was fit to a single-exponential curve with a constant of 0.75 ± 0.03 pN. *N* and *R*^2^ in panels B-F indicate the number of measurements and the square of the regression error, respectively.

To understand the relationship between the mechanochemical reactions of monomeric and dimeric dynein molecules, we evaluated the reaction rate, force and duty ratio of dimeric dynein. A dimeric dynein (GST-D384) was constructed by fusing a glutathione S-transferase (GST) tag to the N-terminus of D384. We measured the force and displacements of single molecules of GST-D384 bound to single trapped beads at 1 mM ATP. Dimeric dynein took stepwise movements up to a force of 2.5 pN toward the minus end of the microtubule (Figs 5A and S6). The predominant step size at low loads (<0.5 and 0.5–1.1 pN) was ~16 nm, and the minor steps were ~8 nm (Fig. 5B). The step size of ~8 nm became predominant at a force of 1.1–2.5 pN (Fig. 5B). The histogram of the dwell time fit well to single-exponential curves, with a time constant of 45.1 ± 0.4 ms, 68.6 ± 0.4 ms and 101 ± 1 ms in the force ranges of <0.5, 0.5–1.1 and 1.1–2.5 pN, respectively (Fig. 5C). The stepping rate, the inverse of the time constant, decreased exponentially with the force (Fig. 5D). The stepping rate extrapolated to no load was 25.3 ± 0.2 s^−1^.

To obtain the force-velocity relationship, the velocity was estimated by the mean step size divided by the mean dwell time (Fig. 5E). By extrapolating the linear relationship, the maximum velocity and force were found to be 387 ± 5 nm s^−1^ and 1.85 ± 0.01 pN, respectively. The occurrence frequency of the peak force or peak displacement measured immediately before dynein dissociated from the microtubules decreased exponentially with a force constant of 0.75 ± 0.03 pN (mean ± s.e.m.) or displacement constant of 47 nm, consistent with the previous report^24^ (Fig. 5F). This result indicates that the number of dynein molecules interacting with microtubules decreased to the inverse of exponential (e^−1^) per 3.3 steps or e^−1/3.3^ (=0.74 ± 0.02) per step. In other words, 26% (1–0.74) of dynein molecules were dissociated from the microtubules at each step. Because both heads should be dissociated from the microtubule upon dissociation of dimeric dynein, the duty ratio *r*_d_, which is the fraction of time that each head of dynein spends attached to a microtubule, was calculated to be 0.49 ± 0.01 from the equation (1-*r*_d_)^2^ = 0.26, assuming independence of the reactions between the two heads.

The velocity (387 nm s^−1^, Fig. 5E) of single molecules of dimeric GST-D384 at no load was significantly slower than the microtubule gliding velocity (481 ± 8 nm s^−1^ at 1 mM ATP) measured by the *in vitro* motility assay, in which many GST-D384 molecules interacted with microtubules. The velocity difference can be explained by the duty ratio of dimeric dynein. The duty ratio (*r*_d_) of each dynein head was calculated to be 0.59 ± 0.02 from the equation *V*_2_ = *V*_∞_{1 - exp(-2*r*_d_)}/{1 - exp(-2)}, where *V*_2_ (=387 nm s^−1^) is the velocity of a single GST-D384 molecule, and *V*_∞_ (=481 nm s^−1^) is the sliding velocity obtained by the *in vitro* motility assay at 1 mM ATP^25^. The duty ratio (0.59 ± 0.02) of each head in dimeric dynein obtained here was close to that (0.49 ± 0.01) calculated from the run length. These ratios are consistent with the reported value (0.58) of the *Dictyostelium* dimer^25^.

## Discussion

This study quantified the ATP-dependent mean displacement of single-headed human dynein using the dumbbell method of optical tweezers. The stroke size was calculated to be 8.3 nm at saturated ATP concentrations. The step was driven by the power stroke because the mutant without linker swing did not take steps. The transition rate from the MD-pre to the MD-apo state calculated from the binding times was not much different from the dissociation rate of MD-apo to D-apo under no load. Starting from these mechanical and chemical data, we discussed the mechanochemical reactions through which dynein interacts with microtubules.

### Power stroke size

We succeeded in measuring the displacement of a single-headed cytoplasmic dynein and determined its mean displacement by changing the ATP concentration in the range of 0–100 μM. To explain the ATP-dependent displacement, we also investigated the efficiency of FRET from ring-BFP to linker-GFP depending on the ATP concentration. From these results, we proposed that the population of dynein in the prestroke and poststroke states binding to microtubules respectively decreased and increased as the ATP concentration decreased. Based on this proposition, we calculated the rate constants of the main chemical cycle of dynein. The *K*_m_^E^ value was 3.3 ± 0.3 μM, which was close to that (1.8 μM) obtained by the FRET measurement of *Dictyostelium* dynein^19^. The binding ratio (*k*_apo_^on^/*k*_pre_^on^) of dynein was initially calculated to be 5.3 ± 0.5, which indicates that dynein bound to microtubules 5.3 ± 0.5 times faster in the D-apo state than in the D-pre state. The ATP-dependent displacements found in dynein have not been reported in single-headed kinesin and myosin^26,27^ because the *K*_m_^E^ of myosin S1 and kinesin-1 were over 100 times smaller (only ~10 nM) than those of dynein-1^26,28,29^, and their *K*_m_^d^ values were much smaller than those of dynein as well.

The power stroke size (8.3 ± 0.3 nm) of a single-headed dynein at a saturated ATP concentration was compared with the structural evidence. The stroke size of 8.3 nm was within the range of the ring shift, as previously reported by EM observations^9–11^ (Table S1). Recently, it was reported that the head of dimeric dynein moves forward without largely changing the angle between the stalk and the axis of the microtubule^30^. This result suggests that a head of dynein can undergo a translational motion of 8.3 nm without appreciably rotating the stalk.

### Reaction rates in the model

The reaction rates in the model were determined by the analysis of microtubule binding time and biochemical assay. The microtubule binding time was not highly dependent on ATP concentration (Figs 4 and S2C). The mean binding time (equation 11) calculated from equation 10 was also not highly dependent on the ATP concentration (Fig. S3D). This is because the dissociation rate (8.3 s^−1^) of dynein from the microtubule at the MD-apo state was close to the rate (*k*_M1_ = 12 s^−1^) at high ATP concentrations.

A second-order rate constant (*k*_MT_ = 0.29 ± 0.10 μM^−1^s^−1^) of ATP binding was smaller than that (2–4 μM^−1^s^−1^) of axonemal 22 S (measured at 28 °C) and *Dictyostelium* cytoplasmic dynein^17,19^. The discrepancy between the present and previous values can be explained by the difference of the species, or that the reverse reaction rate of ATP binding (*k*_-MT_, from the MD-ATP to MD-apo state) is compatible with the dissociation rate (*k*_ATP_^off^) from the MD-ATP to D-ATP state. In the latter case, the rate constant (*k*_MT_) of ATP binding is given by (*k*_ATP_^off^*k*_MT_/*k*_-MT_). *k*_MT_ becomes small at large *k*_ATP_^off^ /*k*_-MT_.

The dissociation rate (*k*_apo_^off^) from MD-apo to D-apo was calculated to be 8.3 ± 0.4 s^−1^ under no load. The unbinding rates of yeast dynein were ~2 s^−1^ at loads of 1 and −1 pN in no-nucleotide conditions^31^, which is the same order as the present value. On the other hand, the unbinding rate of single-headed kinesin-1 and myosin-V at no load was very low (~0.007 s^−1^)^32,33^, indicating that the dissociation rate (*k*_apo_^off^) of dynein was much faster than that of kinesin-1 and myosin-V.

The binding rate (*k*_apo_^on^) from D-apo to MD-apo was 5.5 μM^−1^s^−1^, as calculated from *k*_apo_^off^ and the dissociation constant (*K*_d_^apo^) (Fig. S5). It is larger than that obtained by axonemal dynein (1.6 μM^−1^s^−1^)^18^. In the case that one head of dimeric dynein dissociates from microtubule in the MD-apo state, the head rebinds very quickly to the microtubule because the tubulin concentration around the unbound head is very high (~1 mM). This suggests that the dissociation rate of dimeric dynein from microtubule is much lower than that of monomeric dynein. This suggestion is supported by the result that the *K*_m_^MT^ of dimeric dynein is much smaller than that of single-headed dynein (Table S2)^34,35^.

The rate *k*_pre_^on^ from the D-pre to the MD-pre state was calculated to be 1.0 μM^−1^s^−1^ from *k*_apo_^on^ divided by *k*_apo_^on^/*k*_pre_^on^ (=5.3) (Tabel 1). The transition rate *k*_pre_^off^ from the MD-pre to the D-pre state was estimated to be 34 s^−1^ from *k*_pre_^on^ multiplied by the dissociation constant (*K*_d_^Vi^), assuming that the ADP-Vi state mimics the prestroke state (Fig. S5). The lower affinity of dynein to the microtubule in the prestroke state was realized by the higher dissociation rate (*k*_pre_^off^) and lower association rate (*k*_pre_^on^) when compared to those at the apo-state.

*k*_M1_ (12 ± 1 s^−1^) acts as a rate-limiting step of the ATPase reaction at high concentrations of polymerized tubulin and ATP, because the rates from MD-apo to D-pre via the MD-ATP state at high ATP concentrations were much faster than *k*_M1_ (Fig. 2)^17,19,20,23^, and the binding rate (*k*_pre_^on^) is fast at high concentrations of microtubules. The value of *k*_M1_ was within the error of the maximum turnover rate (*k*_cat_, 9.3 ± 2.5 s^−1^), which was close to that in previous reports^36–40^ (Table S2).

### Step size and reaction rates of dimeric dynein

The traces of force generation of double-headed dynein showed various shapes, which were similar to previous studies (Figs 5A and S6)^39,41^. The occurrence fraction of force between 2.0 to 2.5 pN was only 2.7% in total (Fig. 5F), consistent with previous studies (3% over 2.1 pN force from the Nicholas and Tripathy group)^39,41^. The maximum force of 1.85 pN was consistent with previous studies^2,38–43^ and was lower than that in other studies that included dynein adapters^3,43–45^ (Table S3). One reason for the low force generation is that the mammalian dynein head had a limited duty ratio (*r*_d_, 0.49–0.59) to move continuously along the microtubule, resulting in the dissociation of dynein from the microtubule before dynein generated the stall force. In the recent studies, when mammalian dimeric dynein interacts with dynactin and Bicaudal D (or other adapters), dynein disrupts its autoinhibited form and increases its affinity to microtubules. The dynein complex moves in a highly processive fashion, and therefore generates a larger stall force than dimeric dynein^40,43–47^.

The population of dynein that took large steps (~16 nm) decreased at the higher load, consistent with previous reports^2,48^ (Fig. 5B). At a low load (<0.5 pN), the step sizes of double-headed dynein were ~8 and ~16 nm, consistent with previous studies^2,4–6^ (Fig. 5B).

The distribution of the dwell time of dimeric dynein decayed single-exponentially at 1 mM ATP, suggesting that dynein has one rate-limiting state with an ATP turnover rate (*k*_cat_). The rate (25.3 ± 0.2 s^−1^, Fig. 5E) of stepping extrapolated to no load of dimeric dynein was close to previous reports (37 s^−1^)^3^. The rate (25.3 ± 0.2 s^−1^) was not significantly different from double (18.6 ± 5 s^−1^) of the ATP turnover rate of a single-headed dynein (D384), supporting a tight coupling model of one step per ATP molecule hydrolysis at the AAA1 ATPase site, because the turnover rate per double-headed dynein was close to twice that of a single-headed dynein^14,15^. Therefore, the turnover rate of monomeric dynein D384 is reasonable in comparison with the stepping rate of a dimeric dynein under no load.

### Walking model of dimeric dynein

**Figure 6:**
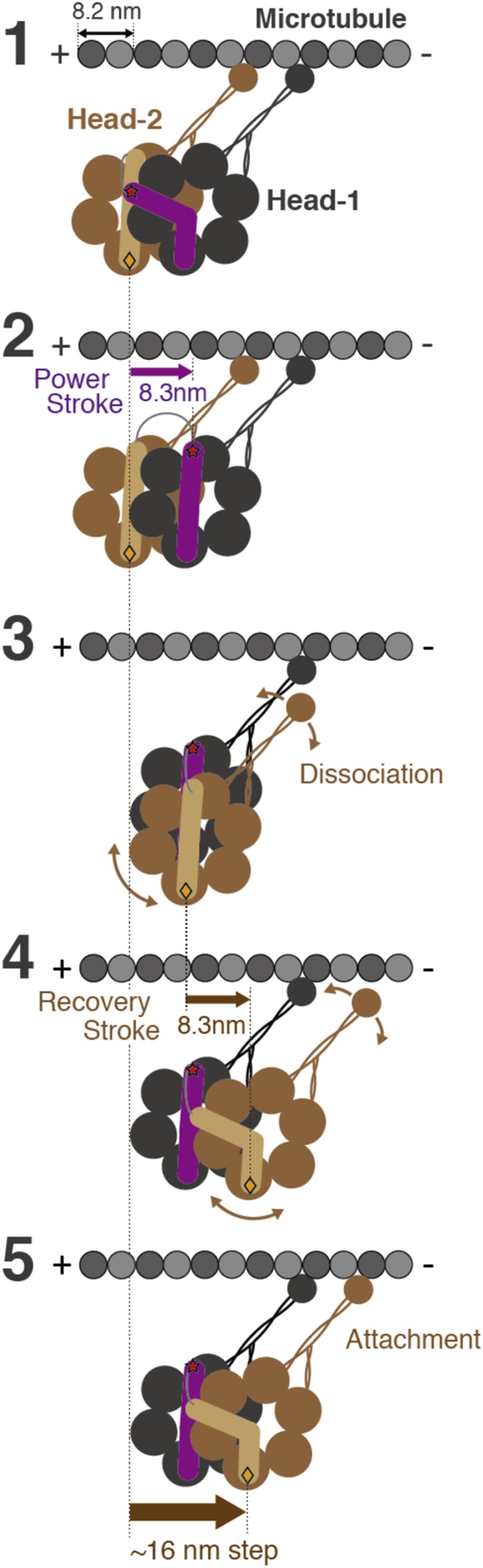
Walking model of dimeric dynein with ~8-nm step during single ATP hydrolysis at the AAA1 catalytic site. After stage 3, head-2 is shown in front of head-1 to trace its motion easily.

Based on the present results of single- and double-headed dynein molecules, we propose a walking model of dimeric dynein to address the contribution of the power stroke of 8.3 nm to taking steps (Fig. 5). In the model, dimeric dynein has five stages during the hydrolysis of single ATP molecules at the AAA1 catalytic site (Fig. 6). The leading head-1 of dimeric dynein generates a power stroke of 8.3 nm (stage 1 to 2). Head-2 takes the load caused by the intramolecular strain between head-1 and head-2, and the dissociation of head-2 from the microtubule is then accelerated by the intramolecular tension (stage 2 to 3). The position of the stalk in dissociated head-2 will be close to that in head-1, as supported by electron microscopy images of the superposed stalk-heads observed in the poststroke state (stage 3)^49^. Head-2 moves forward according to a recovery stroke of 8.3 nm (stage 4)^9,30^ and subsequently attaches to the microtubule (stage 5). In this way, one head of dynein takes an ~16 nm step, or the center of the dimer heads take an ~8 nm step, which is the predominant step size under high load. The 16 nm step of the head is predominantly generated by the power stroke and recovery stroke. From stage 4 to 5, head-2 makes a diffusive search for the next microtubule binding site, and therefore dimeric dynein is able to take a large step (16 nm), as observed for double-headed dynein, for which even the stroke size is consistently 8.3 nm. In the present model, the biased Brownian motion that significantly contributes to the stepping of kinesin-1 and myosin-V^27,50^ is not necessary for an 8 nm-step of dynein. Our new findings regarding single-headed dynein are basic and crucial to understanding the molecular mechanisms underlying the motility of dimeric dynein, including the full-length of dynein and its complexes.

## Materials and Methods

### Preparation of recombinant human dynein

The cDNA (KIAA0325) of human cytoplasmic dynein-1 was obtained from the Kazusa DNA Research Institute (Japan). We created the human cytoplasmic dynein monomer construct D384, with cDNA encoding Gly^1288^-Glu^4646^ (384 kDa), that was subcloned into the pFastbac1 vector (Invitrogen) (Fig. 1A). A FLAG-tag and a biotin-carboxyl carrier protein (BCCP) were inserted at the N-terminal end of the dynein motor domain. D384, a single-headed construct, includes a short tail (Gly^1288^-Val^1319^) without the ability to dimerize. For fluorescent measurements, AcGFP (Clontech) was inserted between the BCCP and dynein motor domains, and pRSET/BFP (Invitrogen) was inserted between Glu^2404^ and Gly^2405^ (D384GB) (Fig. 1A and C)^12^. In the two mutant constructs (D384GB-ΔPSI and D384GB-ΔPSIΔH2), Leu^2324^-Leu^2335^ (PS-I insert in AAA2 domain) and/or Thr^2267^-Asp^2277^ (H2 insert in AAA2 domain) were replaced by Gly-Gly from the D384GB construct (Fig. S1A)^12^. For artificially dimerized construct (GST-D384), a glutathione S-transferase (GST) tag was inserted between BCCP and the dynein motor domain.

To express dynein, Sf9 cells were infected with viruses expressing the dynein construct according to the Bac-to-Bac system protocol (Invitrogen). The cells were cultured at 27 °C in 175-cm^2^ flasks and then harvested after 72 hours. The cells were lysed and centrifuged at 14 k rpm (17,800 × g) for 20 min, and the supernatant was loaded onto an anti-FLAG M2 affinity gel column (Sigma-Aldrich) over 1 hour. The column was washed, and the dynein was eluted with dynein elution buffer (25 mM PIPES (pH 7.2), 150 mM KCl, 2 mM MgSO_4_, 1 mM EGTA, 5 mM 2-mercaptoethanol, 0.01 mg/ml leupeptin, 0.5 µM ATP, and 0.1 mg/ml FLAG peptide (Sigma-Aldrich)). The peak fractions of the wild-type construct were collected by microtubule affinity in 100 μM ATP, followed by with ultracentrifugation at 80 k rpm (235,000 × g) to exclude the inactive dynein and aggregates. The active dynein in the supernatant was preserved with 10% (w/v) trehalose. The proteins were quickly frozen in liquid nitrogen and were stored at −150 °C. The protein concentrations were determined by densitometry of an SDS-PAGE gel stained by Coomassie Brilliant Blue using ImageJ (NIH), or they were measured by the Bradford protein assay (Thermo) (Fig. S1B).

### Observation of the negative-staining electron microscopy of dynein

D384 (16 nM) in buffer (25 mM PIPES, pH 7.2, 25 mM K-acetate, 2 mM MgCl_2_, and 1 mM EGTA) was applied to carbon-coated copper grids and negatively stained with 1% (w/v) uranyl acetate. We observed the samples under a Tecnai F20 electron microscope^9^.

### Preparation of microtubule and mutant kinesin

We constructed a mutant of the double-headed kinesin-1 (KIF5A) from mouse for adhesion between the beads and microtubules. BCCP and the His-tag were fused at the C-terminal of kinesin (K490, Met^1^-Ala^490^). We used point-mutated G235A in K490, which does not dissociate from the microtubule in the presence of ATP^51,52^. Kinesin was purified by a previously reported method^53^. Briefly, the kinesin was purified by a Profinity IMAC Ni-charged resin affinity chromatography column (Bio-Rad) (Fig. S1B). The purified kinesin was frozen in liquid nitrogen and stored at −80 °C.

Tubulin was purified by a previously reported method^14^. Tubulin was labeled with TAMRA (carboxy-tetramethyl-rhodamine succinimidyl ester), and the polarity-marked microtubules were prepared as previously described^54,55^. The correctness of the orientation of the polarity-marked microtubules was determined to be 93% by the direction of the microtubule gliding. The concentration of proteins was measured with a Bradford protein assay (Thermo).

### FRET efficiency measurement

Samples containing 100 nM dynein in assay buffer were excited at 380 nm, and the emission spectrum was scanned from 400 to 600 nm using a spectrofluorometer^14^ (FP-6600, Jasco). The FRET efficiencies were calculated using the reported equation^14^. The molar extinction coefficients were 32,500 and 2,810 M^−1^cm^−1^ for GFP at 488 and 380 nm, respectively, and 27,750 M^−1^cm^−1^ for BFP at 380 nm. In Fig. 1D, the efficiencies of FRET were measured at the nucleotide concentration of 1 mM (for ADP-Vi, 0.2 mM ADP plus 1 mM vanadate). In Fig. 1E, the efficiencies were measured in the presence of ATP and an ATP-regenerating system, such as 10 mM creatine phosphate and 10 units/ml creatine phosphate kinase (Sigma-Aldrich), to keep the ATP concentration constant. The curve fitting of ATP-dependent efficiency was performed by graphic software (KaleidaGraph, Hulinks Inc.).

### Protein-coated beads

Polyethylene fluorescent beads (Molecular Probes, yellow-green, 200 nm in diameter, and blue fluorescence, 1 μm) were bound to NeutrAvidin (Thermo) by incubating them in a solution of EDC (Thermo) and sulfo-NHS (Thermo)^56^. For the dumbbell assay, the avidin-coated beads, 1 μm and 200 nm in diameter, were incubated with biotinylated dynein and mutant kinesin, respectively, for 20 min at room temperature. Approximately 25% of the dynein-coated beads interacted with the microtubules. The probability of two or more molecules interacting with the microtubules simultaneously was calculated to be < 0.019 of the binding events; thus, we measured single molecules in almost all experiments. For the single-trap assay, the binding fraction of dynein-coated beads (200 nm) to microtubules was approximately 0.2. The probability of two or more molecules interacting with the microtubules simultaneously was calculated to be <0.014 of the binding events.

### Single-molecule assay of monomeric dynein by optical tweezers

A custom-made microscope system for the optical tweezers was used for dumbbell and single trapping experiments, as previously described^56,57^. Dynein-coated beads were fixed on the glass chamber and were washed 4 times by solution exchange. The microtubules that bound to the trapped beads (200 nm in diameter) in the flow chamber were brought into contact with the dynein attached to the dynein-coated beads (1 μm in diameter) in assay buffer (25 mM PIPES, pH 7.2, 25 mM K-acetate, 2 mM MgCl_2_, 1 mM EGTA, 0.2 mg/ml casein, 10 μM paclitaxel and an oxygen scavenger (20 mM glucose, 20 μg/ml catalase, 0.1 mg/ml glucose oxidase and 140 mM 2-mercaptoethanol) at 25 ± 0.5 °C) (Fig. 3A). The final contaminating concentration of ATP and GTP should be <10 nM and ~220 nM, respectively. It has been reported that 22 S dynein did not dissociate from microtubules in the presence of 0.5 mM GTP^58^; therefore, the contaminating ATP and GTP should have a negligible effect on the binding experiment, even in the absence of ATP. In the experiment of D384, the ATP concentration ranged from 0 to 100 μM, because the binding period over 50 ms was not well detected above 100 μM ATP. In the experiment of D384GB-ΔPSIΔH2, assay buffer containing 150 mM K-acetate was used to accelerate the dissociation rate because the mutant bound to microtubule for >1 min in the assay buffer containing 25 mM K-acetate, even in the presence of 1 mM ATP.

The trap stiffness was determined from the variance of position of the trapped bead (16 fN/nm) due to thermal fluctuation^3^. The stiffness in the sampling window of 24 ms was calculated successively by shifting the half window (12 ms) of the acquired data using a customized MATLAB program^21,59^. We considered dynein to be bound to the microtubules for a period of over 48 ms when the stiffness became >52 fN/nm, which was approximately 4 times the stiffness at no interaction. The displacement of dynein was calculated from that of the trapped beads with an attenuation factor (1.20 in the mean) to account for the compliances of kinesin-bead and dynein^60^. The total numbers of the detected displacements and dynein-coated beads were 470–850 and 4–12, respectively, in each nucleotide condition. The analyses were performed using graphical software (KaleidaGraph, Hulinks Inc. and Origin, OriginLab).

For single-bead trapping, biotinylated polarity-marked microtubules were fixed to streptavidin, which bound to biotinamidocaproyl-BSA on the glass surface. A dynein-coated bead was trapped by optical tweezers and then brought into contact with the microtubules (Fig. S2B). The trap stiffness was 8.1 fN/nm. The stiffness upon dynein binding to microtubules increased to >22.3 fN/nm.

### Single-molecule assay of dimeric dynein by optical tweezers

To detect the stepping motion of dimeric dynein (GST-D384), we conducted a single-molecule assay by using a single trap. Cover glasses were washed with a plasma cleaner (PDC-32G, Harrick Plasma), treated with amino-silane (KBE-603; Shin-Etsu Silicone) and glutaraldehyde (Wako)^61^, and fixed with polarity-marked microtubules with casein blocking. A dynein-coated bead was then trapped with a trap stiffness of 16 fN/nm and was brought into contact with microtubules in the presence of 1 mM ATP. We analyzed the stepping events by a step-finding algorithm using the MATLAB program^62^. Histograms of the step size and those of the dwell time (Fig. 5B and C) were included only before dynein generated the peak force.

### *In vitro* motility assay

Motility assays of dynein and kinesin were carried out by a previously described method with modifications^63^. The solutions of 2 mg/ml biotinamidocaproyl BSA (Sigma-Aldrich), 1 mg/ml streptavidin (Wako), ~100 nM biotinylated dynein, and fluorescently labeled microtubules were sequentially applied to a flow chamber. The velocities of the microtubules in the assay buffer were determined by fitting fluorescence spots to a two-dimensional Gaussian distribution using customized software (kindly provided by K. Furuta, NICT, Japan)^40^. The velocity of monomeric dynein (D384) was 432 ± 11 and 474 ± 10 nm/s at 1 and 2 mM ATP, respectively. The velocity of D384GB was 229 ± 3 and 195 ± 3 nm/s at 1 and 2 mM ATP, respectively. The velocity of dimeric dynein (GST-D384) was 481 ± 8 and 656 ± 12 nm/s at 1 and 2 mM ATP, respectively. These data confirmed that the activity of constructs was retained^34–42,64–67^ (a list of the previous reports from various species is given in Table S2). As shown in Fig. S1C, the microtubules glided at a steady speed without stop-and-go behavior, indicating that the inactive dynein was not included.

### ATPase assay

The basal ATPase rates (*k*_basal_) in the absence of microtubules and the microtubule-stimulated ATPase rates (*k*_cat_) of single-headed dynein D384 were measured by an EnzChek phosphate assay kit (Molecular Probes) by following the method of Kon and colleagues^34^. We mixed 35 nM dynein (D384 or D384GB), 0–25 μM microtubules, 200 μM 2-amino-6-mercapto-7-methylpurine riboside and 0.2 U of purine nucleoside phosphorolysis, followed by the addition of 1 mM ATP before measuring the absorbance at 360 nm at 25 ± 0.5 °C. The ATPase rates were determined by fitting the data to the Michaelis-Menten equation (Fig. S4).

### Cosedimentation assay

To quantify the affinity between dynein and the microtubules in the pre (ADP-Vi) and apo states, we performed a cosedimentation assay following the method of Imamura and colleagues^20^. We mixed 200 nM GFP-fused dynein (D384G), 0–20 μM microtubules and 200 μM ATP plus 1 mM vanadate or no nucleotide and then incubated them for 10 min, followed by centrifugation at 80 k rpm (245,000 × g) for 10 min. The microtubules were precipitated completely, and the dynein bands were clearly observed by SDS-PAGE (data not shown). The supernatant containing free dynein was collected, and oxygen scavengers were added to the supernatant to measure the fluorescence intensity of AcGFP at 505 nm by spectrofluorometry (FP-6600, JASCO). The bound ratio (*R*_bound_) of dynein to microtubules was calculated to be, where [MT] is the concentration of microtubules (tubulin), and *K*_d_ is the dissociation constant (*K*_d_^Vi^ or *K*_d_^apo^) of dynein from microtubules in the ADPVi and apo states (Fig. S5).

## Electronic supplementary material

Supplementary information
