# Supplementary material for: Step Sizes and Rate Constants of Single-headed Cytoplasmic Dynein Measured with Optical Tweezers
Source: Sci Rep. 2018 Nov 5;8:16333. doi: 10.1038/s41598-018-34549-7 (PMC6218510; doi:10.1038/s41598-018-34549-7)
Supplement: Supplementary file 1 — Supplementary information [file 41598_2018_34549_MOESM1_ESM.docx]

**Supplemental Information**

**Step Sizes and Rate Constants of Single-headed Cytoplasmic Dynein Measured with Optical Tweezers**

Yoshimi Kinoshita^1^, Taketoshi Kambara^1, 2^, Kaori Nishikawa^1^, Motoshi Kaya^1^ and Hideo Higuchi^1^*

^1^ Department of Physics, Graduate School of Science, The University of Tokyo, 7-3-1 Hongo Bunkyo-ku, Tokyo 113-0033, Japan

^2^ Present address: Center for Biosystems Dynamics, RIKEN, 6-2-3 Furuedai, Suita, Osaka 565-0874, Japan

*To whom correspondence should be addressed.

Hideo Higuchi

E-mail: higuchi@phys.s.u-tokyo.ac.jp

**SI Equations**

**Data analysis of displacement by optical tweezers**

The cumulative distribution function, which is calculated by integrating the Gaussian distribution of step size is given by the equation,

(1)

where *x* is the displacement, *μ* is the center of the distribution, indicating the observed step size, and *σ* is the standard deviation. We set the values at negative and positive infinity of *x* to -δ_2_ and (δ_1_-δ_2_) because the amount of experimental data is finite. Equation (1) was modified by

. (2)

All of the displacement data were fitted by equation (2).

There were two populations of dynein: those that took a power stroke after they bound to microtubules and those that did not. In this case, the step distribution should fit to the double cumulative distribution functions of the stroke size (*μ*=*μ*_max_) and non-stroke size (*μ*=0),

(3)

where *σ*’ is the standard deviation and *R*_M1_ is the ratio of the dynein population binding to microtubules in the pre-stroke state (and generating a stroke) to the total population of dynein. At a *μ*_max_ of 8 nm and changing *R*_M1_ from 0 to 1, the sum of the double Gaussian distribution of the displacement was calculated by equation (3). The sum of the double Gaussian distributions fit very well to the single Gaussian distribution (equation 2) because the stroke size was much smaller than the standard deviation (Figs. S3A and B). *μ* obtained by fitting was directly proportional to *R*_M1_ in equation (3) (Fig. S3C).

**Analysis of the ATP-dependent FRET efficiency**

We explain the ATP dependence of the FRET efficiencies, step sizes and binding time in this and following sections. In the absence of microtubules, the ratio of dynein in the pre-state to the total dynein (*R*_1_) was calculated to be

(4)

where *k*_T_ is the ATP binding rate and *k*_1_ is the rate including the power stroke and release of Pi and ADP (Fig. 2).

The efficiency (*E*) was calculated from the summation of *R*_1_ multiplied by the FRET efficiency of the prestroke state (*E*_pre_) and (1-*R*_1_) multiplied by the efficiency of apo state (*E*_apo_). The efficiency was given by the modified Michaelis-Menten equation

$E=R_{1}E_{pre}+\left(1-R_{1}\right)E_{apo}=\frac{\left[ATP\right]}{K_{m}^{E}+\left[ATP\right]}\left(E_{pre}-E_{apo}\right)+E_{apo}$ (5)

where *K*_m_^E^=*k*_1_/*k*_T_. The ATP-dependent efficiencies of D384GB fit well to equation (5), with a *K*_m_^E^ of 3.3 ± 0.3 μM, a *E*_pre_ of 50.0 ± 4.6% and a *E*_apo_ of 18.9 ± 1.0% (mean ± standard error) (Fig. 1E).

**Analysis of the ATP-dependent step size**

*R*_M1_ is the ratio of the dynein population that binds to microtubules in the prestroke state (and generating a stroke) to the total population of dynein. *k*_pre_^on^ and *k*_apo_^on^ are the binding rates to microtubules in the D-pre and D-apo states, respectively (Fig. 2) The mean displacement (*d*) was calculated from the summation of *R*_M1_ multiplied by the stroke size (*d*_pre_) of dynein bound to microtubules in the prestroke state and (1- *R*_M1_) multiplied by the displacement (*d*_apo_) of dynein bound to microtubules in the apo state. The ratio *R*_M1_ was calculated from the population (*k*_pre_^on^*R*_1_) of dynein bound to microtubules in the prestroke state divided by its total population:

$$d=R_{M1}d_{pre}+\left(1-R_{M1}\right)d_{apo}$$

$$=\frac{R_{1}k_{pre}^{on}d_{pre}+\left(1-R_{1}\right)k_{apo}^{on}d_{apo}}{R_{1}k_{pre}^{on}+\left(1-R_{1}\right)k_{apo}^{on}}$$

$$=\frac{\frac{\left[ATP\right]}{K_{m}^{E}+\left[ATP\right]}k_{pre}^{on}\left(d_{pre}-d_{apo}\right)}{\frac{\left[ATP\right]}{K_{m}^{E}+\left[ATP\right]}\left(k_{pre}^{on}-k_{apo}^{on}\right)+k_{apo}^{on}}+d_{apo}$$

　　$=\frac{\left[ATP\right]}{K_{m}^{d}+\left[ATP\right]}\left(d_{pre}-d_{apo}\right)+d_{apo}$ (6)

where *K*_m_^d^ =*K*_m_^E^*k*_apo_^on^/*k*_pre_^on^. Here, we substituted *d*_apo_ ≈ 0 in equation (6) so that dynein binding in the apo state did not take the displacement. The mean displacements fit well to equation (6) at a *K*_m_^d^ of 17.6 ± 0.5 µM and stroke size (*d*_pre_) of 8.3 ± 0.3 nm (Fig. 3E). The rate (*k*_apo_^on^) of dynein binding to microtubules in the D-apo state was 5.3 ± 0.5 times that (*k*_pre_^on^) in the D-pre state (Fig. 2).

**Analysis of ATP-dependent binding time**

We calculated the rate constants *k*_M1_, *k*_MT_ and *k*_apo_^off^ from the binding time depending on the ATP concentration (Fig. 4). The rate constants from MD-pre to MD-apo, from MD-apo to MD-ATP and from MD-apo to D-apo were defined as *k*_M1_, *k*_MT_ and *k*_apo_^off^, respectively (Fig. 2). The distribution *B*_pre_(*t*) of the binding time from the MD-pre state to the dissociating states (D-ATP and D-apo states) was calculated by double exponentials as follows.

$B_{pre}\left(t\right)=\frac{k_{M1}k}{k-k_{M1}}\left(e^{-k_{M1}t}-e^{-kt}\right)$ (7)

where *k* is (*k*_MT_[ATP] + *k*_apo_^off^) and is not equal to *k*_M1_. When *k*_M1_ is equal to *k*, the distribution *B*_pre_(*t*) is expressed as

$B_{pre}\left(t\right)=k_{M1}^{2}e^{-k_{M1}t}$ (8)

In the other reaction route, dynein binds to microtubules in the D-apo state (MD-apo) and then binds to ATP (MD-ATP) or dissociates from microtubules before ATP binding (D-apo) (Fig. 2). In this case, the distribution *B*_apo_(*t*) of the binding time is calculated by a single exponential, as follows:

(9)

Considering that dynein bound to microtubules in the D-pre and D-apo states, the binding time *B*(*t*) was calculated from the summation of the binding times *B*_pre_ and *B*_apo_ multiplied by the ratios *R*_M1_ and 1- *R*_M1_:

$$B\left(t\right)=R_{M1}B_{pre}\left(t\right)+\left(1-R_{M1}\right)B_{apo}\left(t\right)$$

(10)

, where α is and β is . All of the histograms were fitted globally by equation (10) (solid lines in Figs. 3 and S3C). The obtained best set of parameters (*k*_M1_, *k*_MT_ and *k*_apo_^off^) was 12 ± 1 s^-1^, 0.29 ± 0.10 μM^-1^ s^-1^ and 8.3 ± 0.4 s^-1^, respectively, with a square of regression error equal to 0.95.

The areas of first and second terms of equation 10 were calculated by integration of each term, that is, $\alpha k_{M1}[ATP]\cdot k_{M1}^{-1}$ and $\alpha\beta\cdot k^{-1}$, respectively. The averaged binding time was calculated by the time constants (*k*_M1_^-1^ and *k*^-1^) multiplied by the fractions of the areas;

$\overline{B(t)}=\frac{[ATP]\cdot k_{M1}^{-1}+\beta k^{-2}}{[ATP]+\beta k^{-1}}$ (11)

Appling the best values of parameters (*k*_M1_, *k*_MT_ and *k*_apo_^off^) in this equation (11), the averaged binding time was not dependent so much on ATP concentration (especially under 10 μM), as shown in Figure S3D. This implies that the degree of exponential decay in histograms (Figs. 4 and S2C) was similar.


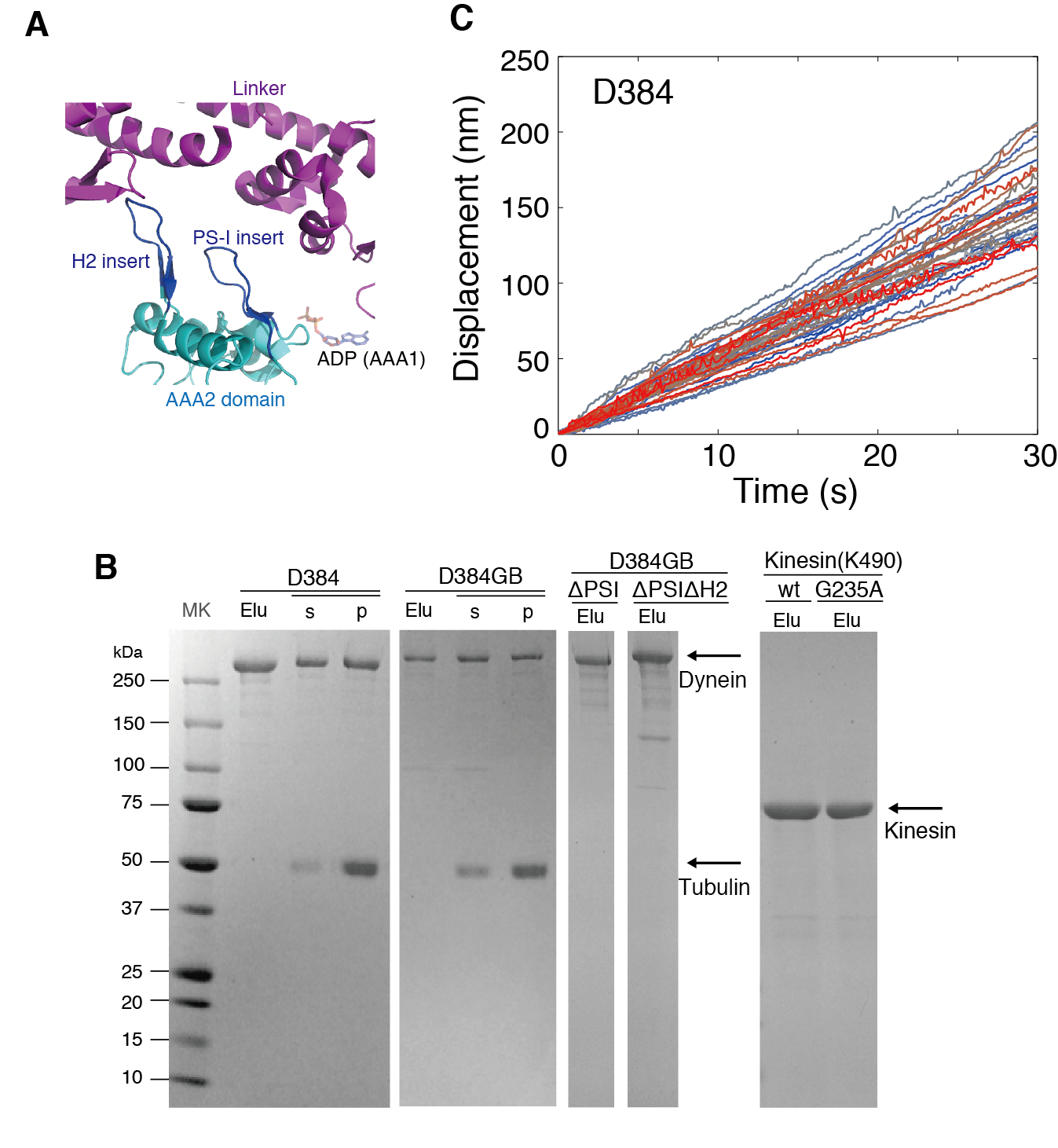


**Figure S1**

Preparation of dynein and kinesin. (A) The crystal structure of dynein motor (edited from PDB 3VKG). The PS-I and H2 inserts in AAA2 domain interact with the linker in the ADP state. The inserts were deleted for mutant dynein in the present study. (B) SDS-PAGE of dynein and kinesin. Elu, elution from anti-FLAG and Ni-charged resins. Purified active dynein in supernatant “s” and microtubule-dynein complex in precipitate “p” collected by microtubule-affinity purification. MK, the molecular weight marker. (C) The example traces of microtubules driven by D384 (38 events) at 1 mM ATP by in vitro motility assay.
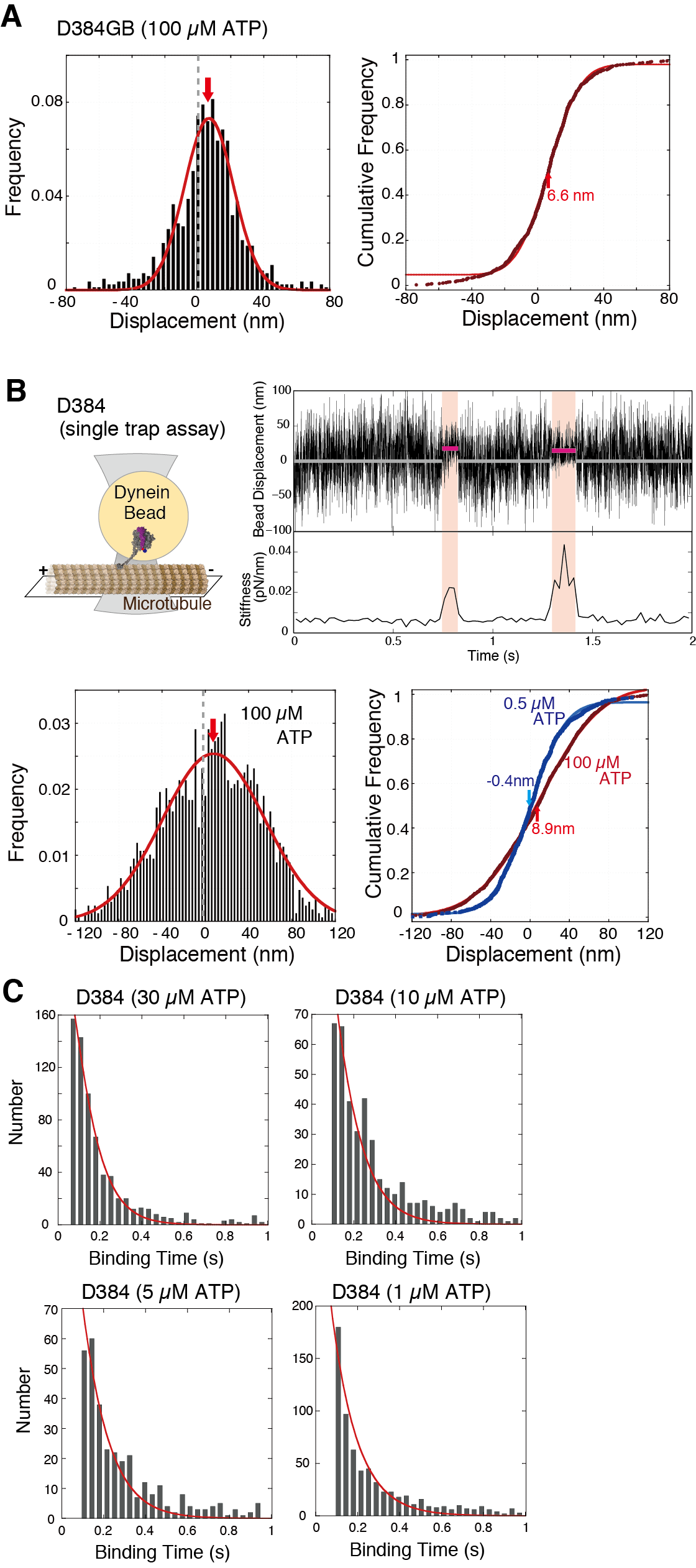


**Figure S2** Displacements and microtubule-binding time. (A) Distributions of displacements of D384GB at 100 *μ*M ATP were measured by the dumbbell assay (849 events, 3 dynein-beads). (B) Upper-left; schematic diagram of single trap assay. Upper-right; time trace of position toward the minus-end of microtubule of the trapped bead coated with D384 at 100 *μ*M ATP. The red zones and lines indicate the period of dynein binding to the microtubule and the displacement during the period at 100 *μ*M ATP. Lower panels; distributions of displacements of D384 at 0.5 *μ*M (blue, 858 events, 10 dynein-beads) or 100 *μ*M ATP (red, 1686 events, 5 dynein-beads) measured by single-trap assay. Left panels in figures A and B show the Gaussian distributions of the displacements. Right panels show the mean displacements determined by fitting experimental displacements to the integrated Gaussian function shown by blue and red lines. Arrows show the mean displacements. (C) Distributions of microtubule-binding time of D384 at 30 *μ*M (740 events, 6 dynein-beads), 10 *μ*M (516 events, 12 dynein-beads), 5 *μ*M (413 events, 4 dynein-beads) and 1 *μ*M (851 events, 8 dynein-beads) ATP. Distributions were fit globally to the equation (10) (red lines). The R^2^ of fitting was 0.98, 0.89, 0.91 and 0.93 at ATP concentration of 30, 10, 5 and 1 *μ*M, respectively.

**
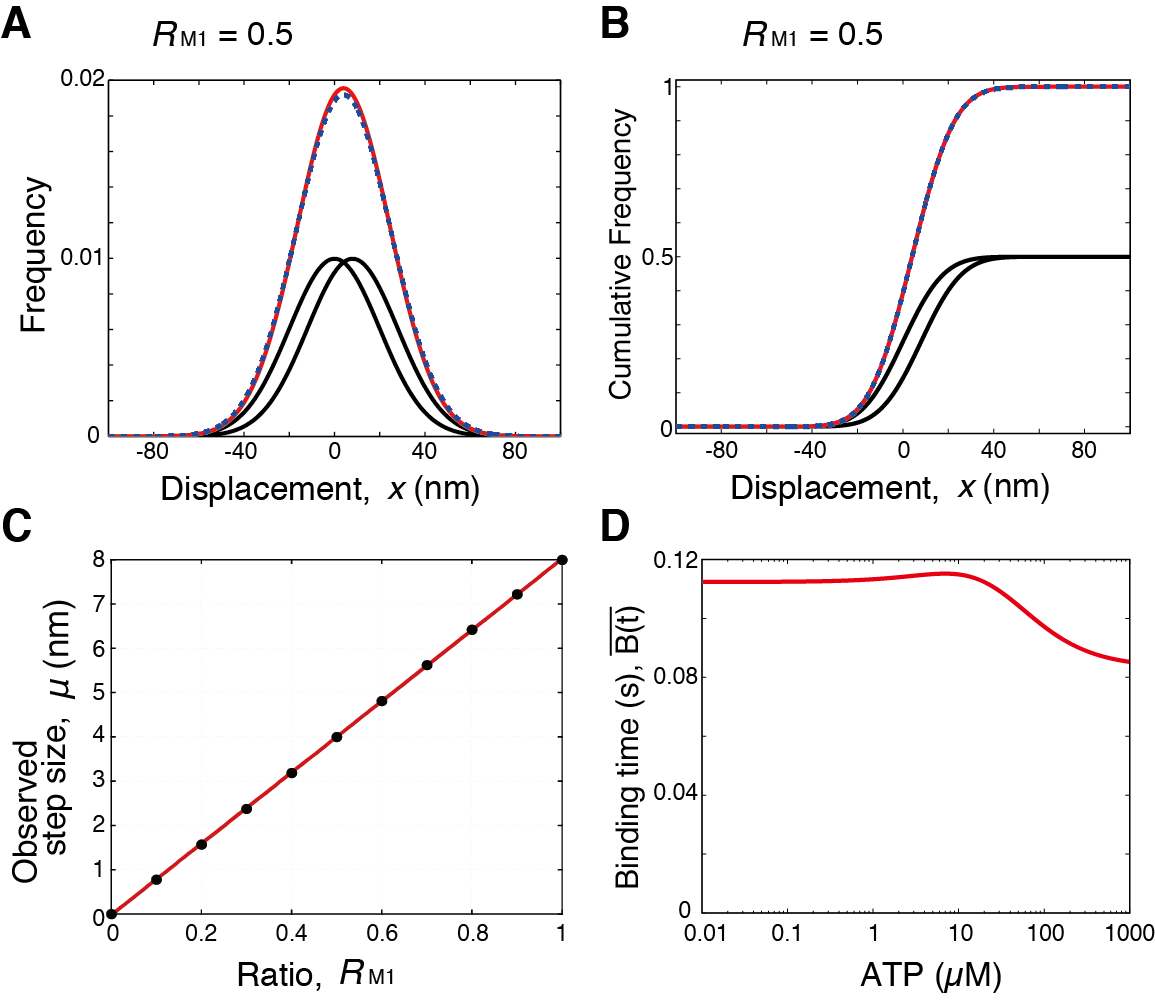
**

**Figure S3**

The step sizes and microtubule-binding time calculated by single distributions and sum of double distributions. (A and B) Black lines show the double Gaussian and double cumulative distributions (equation 1) of dynein binding to microtubules in the pre-state (8 nm) and apo-state (0 nm) at the same probability. Red lines are the sum of the two distribution curves. The red lines fully overlaps with the single Gaussian and cumulative distributions (blue broken lines) at *μ*=4 (nm) in equation (1). (C) The observed step size (*μ* in the equation 1) is almost direct proportional to *R*_M1_ in equation (3) at the stroke size (*μ*_max_) of 8 nm. The regression line was given by the equation, *μ *=8.04*R*_M1_ - 0.02. (D) Microtubule-binding time calculated from equation (10), where the parameters (*k*_M1_, *k*_MT_ and *k*_apo_^off^) of 12 ± 1 s^-1^, 0.29 ± 0.10 μM^-1^ s^-1^ and 8.3 ± 0.4 s^-1^, respectively.


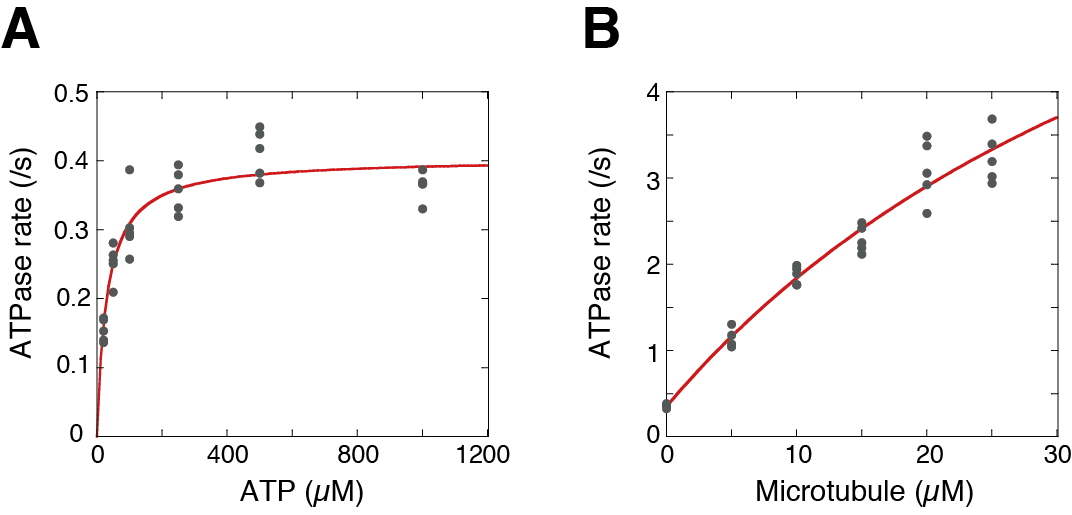


**Figure S4**

ATPase assay of single-headed dynein. (A) The basal ATPase rates of dynein (D384). (B) Microtubule-stimulated ATPase rates of dynein (D384) at 1 mM ATP. The data were fit to Michaelis-Menten equation to obtain the maximum rate (9.3 ± 2.5 s^-1^ of *k*_cat_, 0.40 ± 0.01 s^-1^ of *k*_basal_) and Michaelis-Menten constant (31 ± 4 μM of *K*_m_^ATP^ in figure A, 50 ± 21 μM of *K*_m_^MT^ in figure B).


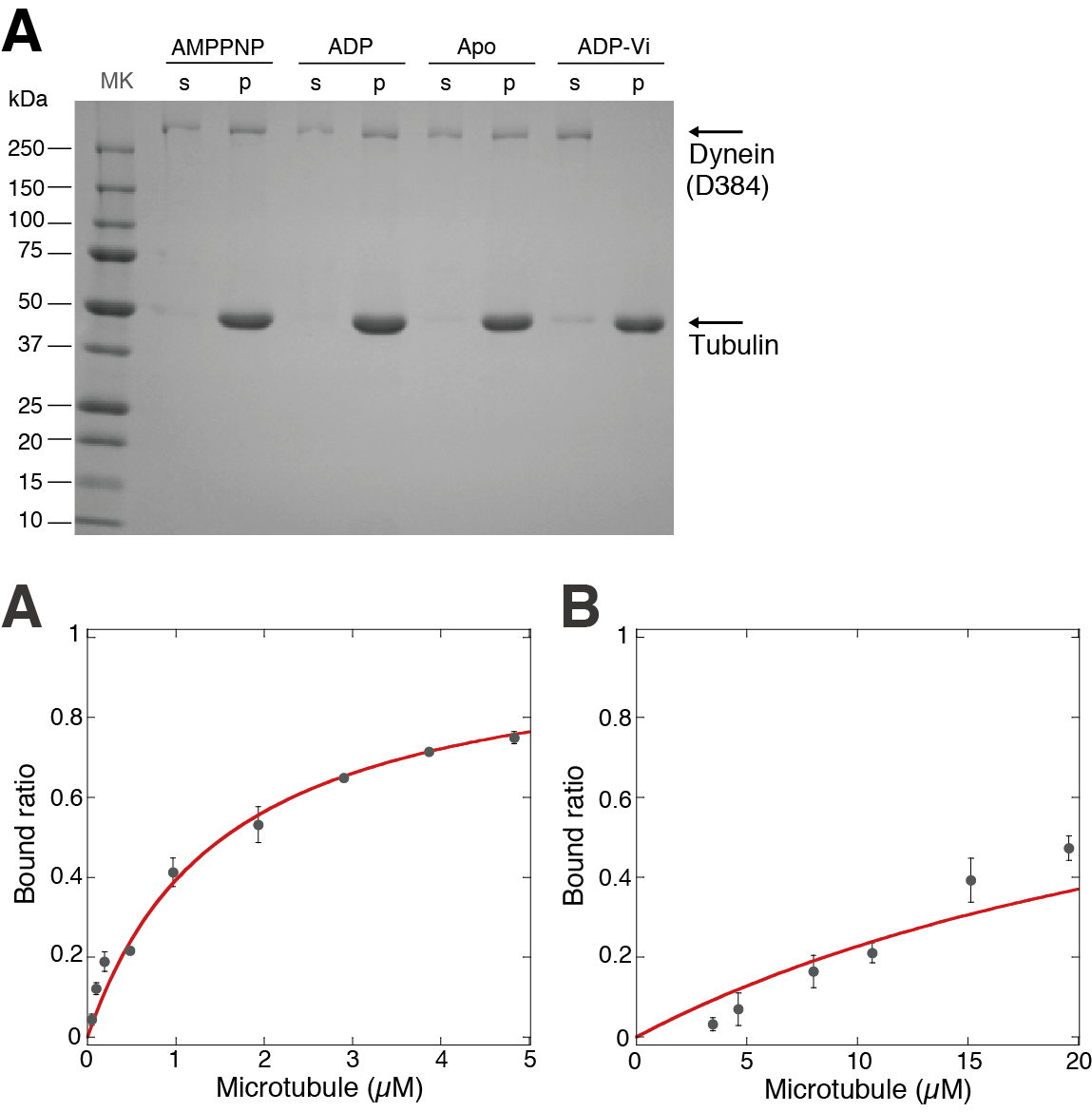


**Figure S5**

Co-sedimentation assay of dynein bound to microtubules. The bound ratio represents the concentration of dynein (D384G) binding to microtubule to the total concentration of dynein in the absence of nucleotide (A) and in the presence of ADP-Vi (B). The data were fit to the Michaelis-Menten equation with the constant of 1.5 ± 0.1 μM in figure A and 34 ± 3 μM in figure B. Bars, standard error.


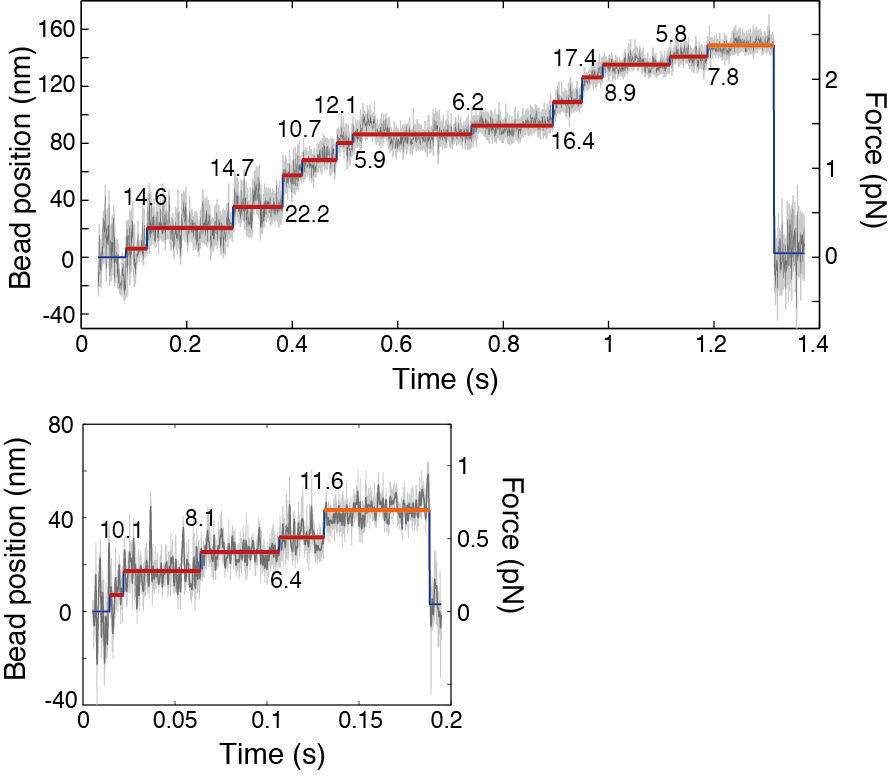


**Figure S6.** Single-molecule movement of dimeric dynein (GST-D384) by the single-trap assay. Displacements of the trap bead of dynein generated largest force at 1 mM ATP. Red lines: dwell time analyzed by the step-finding algorithm. Orange lines: peak force immediately before dynein dissociates from the microtubule. Numbers in figures: the step size was analyzed.

**Table S1** Reported stroke sizes of dynein.


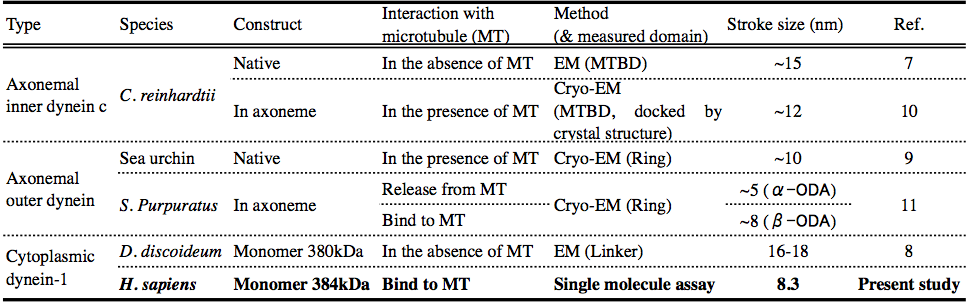


**Table S2** Reported values of reaction rates and microtubule-gliding velocities by in vitro motility assay using single and double-headed dynein.


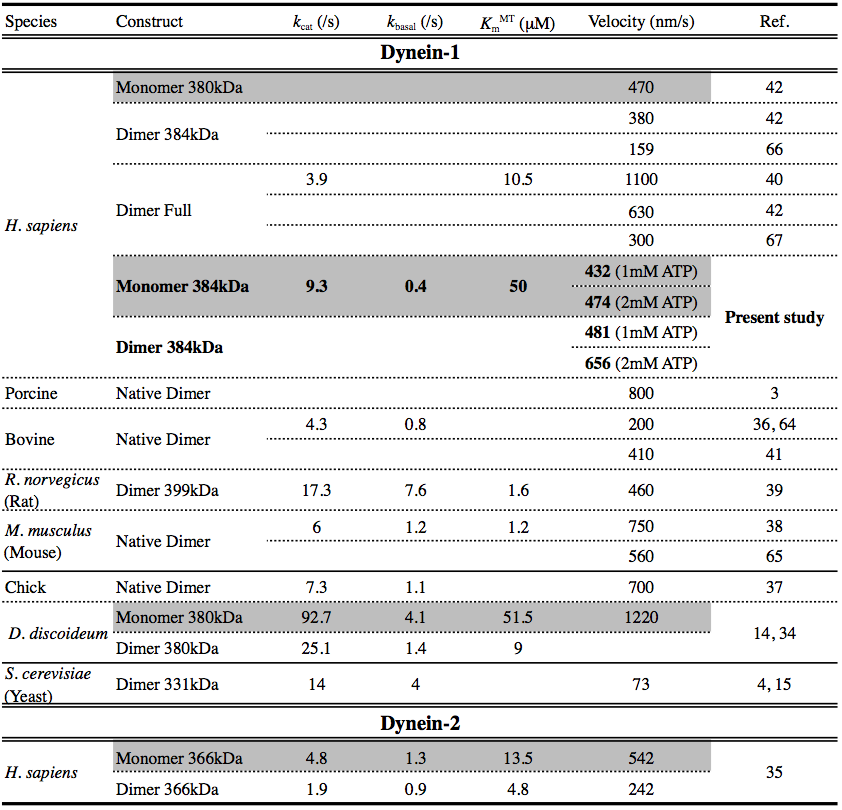


Gray areas indicate the result of monomeric dynein.

**Table S3** Reported stall forces of mammalian dynein or dynein complexes measured by single molecule assay.


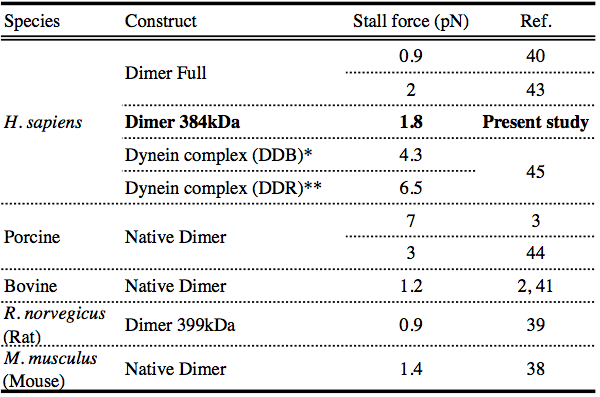


* Dynactin and BICD2 associated with full length of dynein dimer.

** Dynactin and BICDR1 associated with full length of dynein dimer.
